# Comparing two advance care planning conversation activities to motivate advance directive completion in underserved communities across the USA: *The Project Talk Trial* study protocol for a cluster, randomized controlled trial

**DOI:** 10.1186/s13063-022-06746-3

**Published:** 2022-09-30

**Authors:** Lauren J. Van Scoy, Benjamin H. Levi, Cindy Bramble, William Calo, Vernon M. Chinchilli, Lindsey Currin, Denise Grant, Christopher Hollenbeak, Maria Katsaros, Sara Marlin, Allison M. Scott, Amy Tucci, Erika VanDyke, Emily Wasserman, Pamela Witt, Michael J. Green

**Affiliations:** 1 Department of Medicine, Penn State College of Medicine, 500 University Dr., H-041, Hershey, PA 17033-0850 USA; 2 Department of Humanities, Penn State College of Medicine, 500 University Drive, Hershey, PA 17036 USA; 3 Department of Public Health Sciences, Penn State College of Medicine, 90 Hope Drive, Academic Support Building, Suite 2200, Mail Code A210, Hershey, PA 17033 USA; 4 Department of Pediatrics, Penn State College of Medicine, Suite 4400, Mail Code A444, Hershey, PA 17033 USA; 5 Hospice Foundation of America, 1707 L St. NW, Suite 220, Washington, DC, 20036 USA; 6 Department of Health Policy and Administration, The Pennsylvania State University, 604E Donald H. Ford Bldg., University Park, PA 16802 USA; 7 Department of Communication, University of Kentucky, 275 Blazer Dining Hall, University of Kentucky, Lexington, KY 40506 USA

**Keywords:** Advance care planning, Underserved communities, Advance directives, Health games, Terminal illness, Health behavior, Health communication, Mixed methods, Randomized controlled trial

## Abstract

**Background:**

Advance care planning (ACP) is a process involving conversations between patients, loved ones, and healthcare providers that consider patient preferences for the types of medical therapies received at the end of life. Underserved populations, including Black, Hispanic, rural, and low-income communities are less likely to engage in ACP than other communities, a health inequity that results in lower-quality care and reduced hospice utilization. The purpose of this trial is to compare efficacy of two interventions intended to motivate ACP (particularly advance directive completion) for those living in underserved communities.

**Methods:**

This 3-armed cluster, randomized controlled mixed methods design is being conducted in 75 community venues in underserved communities across the USA. The goal of the trial is to compare the efficacy of two interventions at motivating ACP. Arm 1 uses an end-of-life conversation game (*Hello)*; Arm 2 uses a nationally utilized workshop format for ACP conversations (*The Conversation Project*); and Arm 3 uses an attention control game (*TableTopics*). Events are held in partnership with 75 local community-based host organizations and will involve 1500 participants (*n*=20 per event). The primary outcome is completion of a visually verified advance directive at 6 months post-event. Primary analyses compare efficacy of each intervention to each other and the control arm. Secondary mixed methods outcomes include (a) other ACP behaviors and engagement; (b) communication quality; (c) impact of sociocultural environment on ACP (via qualitative interviews); and (d) implementation and sustainability. Subgroup analyses examine outcomes for Black, Hispanic, and rural groups in particular.

**Discussion:**

This trial will add to the evidence base behind various conversational ACP interventions, examine potential mechanisms of action for such interventions, and provide qualitative data to better understand the sociocultural environment of how community-based ACP interventions are experienced by underserved populations. Results will also provide important data for future researchers to learn whether visual verification of advance directives is necessary or whether reliance on self-reported outcomes is of comparable value. Data from this study will inform ways to effectively motivate underserved communities to participate in advance care planning.

**Trial registration:**

ClinicalTrials.gov NCT04612738. Registered on October 12, 2020. All information from the WHO Trial Registration Data Set can be found within the protocol.

## Administrative information

Note: The numbers in curly brackets in this protocol refer to SPIRIT checklist item numbers. The order of the items has been modified to group similar items (see https://urldefense.com/v3/__, http://www.equator-network.org/reporting-guidelines/spirit-2013-statement-defining-standard-protocol-items-for-clinical-trials/__;!!Ls64Rlj6!2NIDdIFnVhEkLOt7JwEBwD7mhpOznkW7beWoypRdL7plaWsG5cmZEkPd4mMC_z1X7ZjrPSsAzbFjYl03oTybArCXEQ$).' above the administrative information table.

**Table:** 

Title {1}	Comparing two advance care planning conversation activities to motivate advance directive completion in underserved communities across the United States: *The Project Talk Trial* study protocol for a cluster, randomized controlled trial
Trial registration {2a and 2b}.	ClinicalTrials.gov NCT04612738. Registered on October 12, 2020.
Protocol version {3}	Protocol version and date. Protocol version 7 dated 6/9/2022.
Funding [1]	This study is supported by the National Institutes of Minority and Health Disparities (R01MD014141). *TableTopics* provided complementary copies of their game for purposes of the study.
Author details {5a}	Van Scoy,^1,2,3^ Levi,^2,6^ Bramble,^7^ Calo,^3^ Chincilli,^3^ Currin,^7^ Hollenbeak,^4^ Grant, ^7^ Katsaros, ^1^Marlin,^,3^ Scott, ^5^ Tucci, ^7^ VanDyke, ^1^ Wasserman,^3^ Witt,^1^ Green^1,2^ ^1^Departments of Medicine, Penn State College of Medicine, Hershey, USA ^2^Department of Humanities, Penn State College of Medicine, Hershey, USA ^3^Department of Public Health Sciences, Penn State College of Medicine, Hershey, USA ^4^Department of Health Policy and Administration, The Pennsylvania State University, University Park, PA, USA ^5^Department of Communication; University of Kentucky, Lexington, USA ^6^Department of Pediatrics Penn State College of Medicine, Hershey, USA ^7^Hospice Foundation of America, Washington, DC, USA
Name and contact information for the trial sponsor {5b}	Not applicable
Role of sponsor {5c}	The funding agency is not involved in the design of the study, data collection, analysis, interpretation of data, writing of the report or decisions to submit reports for publication. The PI is responsible for these activities.

## Introduction

### Background and rationale {6a}

Underserved populations, particularly Black/African American and Hispanic communities, are vulnerable to receiving low-quality care at end of life, resulting in unnecessary physical, psychological, and financial suffering. When compared with white Americans, patients from these underserved communities are (1) 3 times more likely to receive end-of-life care that is not aligned with their preferences [2–5]; (2) significantly less likely to use hospice services at end of life [6–8]; and (3) 3 times more likely to die after a lengthy intensive care unit stay that involves unwanted, burdensome treatments that are unlikely to reduce suffering or improve quality of life [2]. Such health disparities can be addressed in part by advance care planning (ACP)—the process of having conversations with loved ones or clinicians about one’s goals and wishes for end-of-life care and then documenting them in advance directives (ADs), which (for the purposes of this manuscript) includes *both* living wills and healthcare power of attorney documents [9]. Engaging in these ACP behaviors (including the naming of a spokesperson) has been shown to benefit patients, families, and society at large by (1) reducing the incidence of unwanted and aggressive end-of-life medical interventions [10–13]; (2) increasing use of hospice resources [14]; (3) decreasing family members’ anxiety, depression, and post-traumatic stress associated with end-of-life decision-making [12, 15]; and (4) reducing end-of-life costs by up to 36% [16–19].

Efforts to increase ACP and the completion of ADs have included clinical practice guidelines, expert statements, and comprehensive reports documenting the problems with, and need to improve, ACP and end-of-life care in the USA [10, 20–26]. There has been considerable research examining various ACP interventions, ranging from educational activities to multimedia decision aids to one-on-one counseling strategies [27, 28]. Reviews of these research interventions have found heterogeneity of study design, outcomes, and results, which has fueled recent controversy and discourse in the literature questioning the value of ACP itself [29–31].

Nevertheless, by some accounts, these efforts of the past decade have nearly doubled the percentage of white Americans who have engaged in ACP [10]. That said, a systematic review of research studies (comprising 65% white participants) from 2011 to 2016 showed that still only 1 in 3 Americans has completed an AD [32], while other studies have shown that rates of ACP engagement among Black and Hispanic populations have remained consistently lower than 25% [10, 20, 24, 33–38].

In response, there has been a renewed interest in improving opportunities and engagement with the ACP process in underserved communities. In 2021, Jones et al. published a review of empirical studies examining the effect of ACP, palliative care, and end-of-life or terminal care interventions implemented in underserved groups [27]. Of the 16 studies in the review, most were quasi-experimental, and only two were judged to be high-quality randomized controlled trials impacting ACP completion [27]. The first was a randomized controlled trial examining the impact of two didactic, ACP educational sessions for adolescents with HIV [39]. The other trial (involving a sample of veterans in San Francisco) evaluated an online ACP decision aid that included an easy-to-read online AD [40]. Neither of these trials used community-based interventions, and both had geographic shortcomings and niche populations. Several recent systematic reviews have concluded that, although a wide variety of studies have been conducted on ACP interventions, evidence about their efficacy is limited, and findings are mixed [27, 28, 41–43]. Taken together, these findings raise questions about how best to help individuals, particularly minorities, become better prepared for medical decision-making around end-of-life issues.

What is clear, however, is that people living in underserved communities would benefit from engaging in goals of care conversations, including the naming of surrogate decision makers [29, 44–46]. Currently established approaches to ACP fall short because they do not address the two most well-documented barriers to engaging underserved populations: (1) general mistrust of the healthcare system among Black and Hispanic communities [47–51] and (2) a reluctance to discuss death and dying [2, 47, 51–55]. Decades of medical discrimination and misconduct involving underserved populations underlie distrust of the healthcare system, which is often amplified by beliefs that members of these groups are more likely to receive poorer-quality medical care and less truthful information than white Americans [50]. Additionally, reluctance to engage in ACP is driven by a cultural-based discomfort in discussing death and dying within underserved communities [35]. Thus, to make progress toward improving the quality of end-of-life care and reducing unnecessary suffering for underserved populations, affordable, scalable, and effective conversation-based ACP interventions that complement—and do not solely depend upon—encounters with the healthcare system, clinicians, or technology, are needed.

To address these barriers, our team has studied an inexpensive and innovative intervention called *Hello* [56–63] that (1) reframes ACP conversations as a serious game, (2) has proven efficacy to motivate ACP behaviors, and (3) is feasible and scalable at the population level since it does not rely upon one-on-one encounters with healthcare professionals. The game has been found to be effective in motivating ACP across a variety of participant groups, including underserved communities across the USA [64]. Specifically, in multiple studies evaluating the feasibility, acceptability, and efficacy of this game, participants consistently report that *Hello*’s open-ended questions prompt enjoyable and effective discussions of values and preferences for end-of-life care [57–63]. Moreover, after playing *Hello*, 98% of participants performed at least one ACP behavior (e.g., completing an AD, discussing end-of-life issues with loved ones) [57, 58, 60].

In a recently completed study, we partnered with the national education organization, Hospice Foundation of America (HFA), to develop an effective and innovative *community-based delivery model*. Leveraging HFA’s vast network, we recruited community hosts in 53 underserved communities to arrange game events using our materials and protocol. There were 1122 individuals in 27 states who participated in this non-comparative and non-randomized trial. Research assistants traveled to 15 sites in African American communities to collect research data and also completed follow-up phone calls 3 months after the event. We found that that 98% of participants completed at least 1 ACP behavior after the event, 80% discussed their end-of-life wishes with loved ones, and 41% completed a new AD post-event [64].

While the game approach clearly motivates individuals to engage in ACP, and preliminary data suggests that the individuals who do play the game take action by completing an AD, a definitive randomized controlled trial has not been undertaken, nor has the game been compared to other conversation-based ACP interventions.

### Objectives {7}

The overall goal of the cluster, randomized controlled trial described here is to compare the efficacy of two ACP interventions (the *Hello* game and a structured conversation workbook and workshop, *The Conversation Project Starter Guide*) for motivating ACP. The primary outcome is completion of a visually verified advance directive (as opposed to self-reported completion) at 6 months post-event. Analyses compare efficacy of each intervention to each other, and also to a control arm. Secondary outcomes include (a) other ACP behaviors and ACP engagement; (b) communication quality; (c) impact of sociocultural environment on ACP; and (d) implementation and sustainability. There are four specific objectives of the trial:

1. To compare the efficacy of the *Hello* game with *The Conversation Project Starter Guide* and a Placebo/Attention control group—as measured by AD completion and other ACP behaviors. We hypothesize that structuring ACP conversations as a game will motivate greater performance of ACP behaviors within a 6-month follow-up period compared to *The Conversation Project Starter Guide* or a Placebo/Attention control game (*TableTopics*).
2. To evaluate whether conversation quality (measured using Communication Quality Analysis; CQA) serves as a mediator for ACP behavior completion. We hypothesize that high CQA scores will be related to ACP completion.
3. To qualitatively explore how participants’ sociocultural environment affects their experience discussing end-of-life issues during the ACP interventions. These data will be integrated with quantitative efficacy data to investigate potential mechanisms of action for explaining the efficacy of the respective interventions.
4. To explore aspects of intervention implementation by assessing a menu of domains (i.e., intervention characteristics, inner setting) and constructs, grounded on the Consolidated Framework for Implementation Research (CFIR) {Damschroder, 2009 #1958}, and key implementation outcomes (i.e., acceptability, sustainability).

### Trial design {8}

The Project Talk Trial [65] is a 3-armed, cluster, randomized controlled mixed methods, superiority trial delivered in 75 underserved community sites across the USA. The trial compares the efficacy of *Hello* (Arm 1) with a nationally promoted, structured workbook called *The Conversation Project Starter Guide* (Arm 2), and a Placebo/Attention control involving a non-ACP-focused game called *TableTopics* (Arm 3) for motivating various ACP behaviors including AD completion*.* The trial investigates the mechanism of action by which successful ACP interventions motivate underserved individuals to engage in ACP via the secondary outcome measures. The 75 sites are randomized using a 2:2:1 allocation ratio to host events using either the *Hello* game, *The Conversation Project Starter Guide* or *TableTopics* (control) game (respectively) with a goal of 20 participants per site (*n*=1500).

**Fig. 1:**
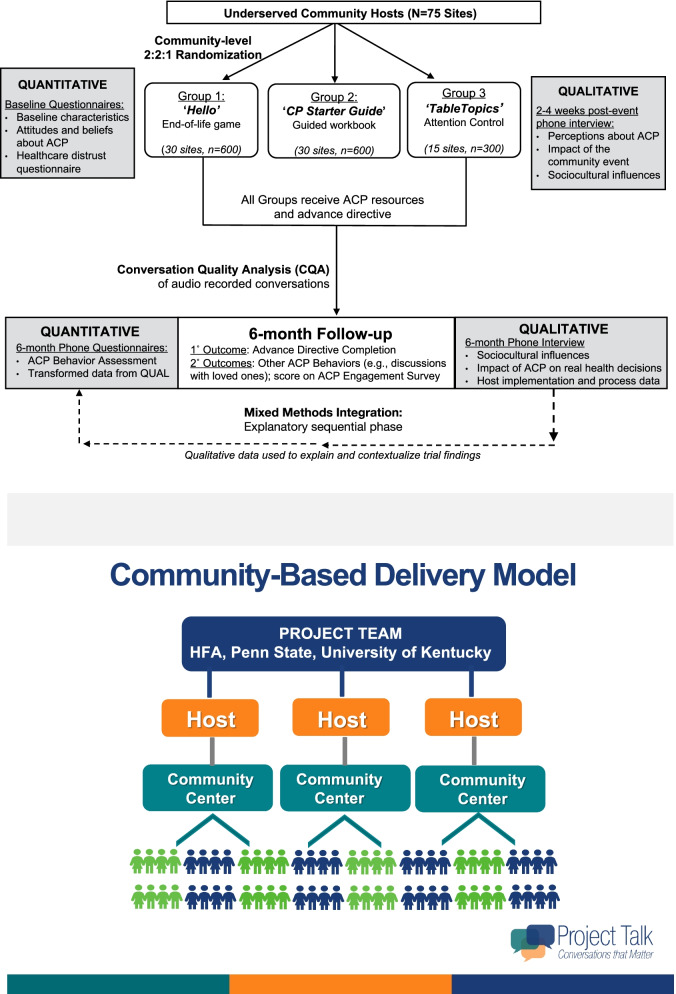
Overview of mixed methods study design. The study design involves a three-armed, cluster randomized controlled trial of two ACP interventions and an attention control. Gray boxes represent mixed methods data elements. CP = Conversation Project; CQA= Communication Quality Analysis; ACP= advance care planning

Using an explanatory-sequential mixed methods design [66], secondary outcomes including qualitative data will explore potential mechanisms of action. Figure 1 illustrates the study design.

## Methods: participants, interventions and outcomes

### Study setting [67]

The study will take place in 75 underserved community venues across the USA (60 of which serve English-speaking communities and 15 serve Spanish-speaking communities). These community events occur in settings like community, senior, and worship centers. Underserved communities are defined using the NIH definition that includes ethnic/racial minority backgrounds, rural ZIP codes, or communities at or below the poverty level based on family size [68, 69]. For this trial, rural sites are defined by the “Am I Rural?” tool from the Rural Health Information Hub that uses zip code to indicate whether communities meet CMS Rural Health Clinics Program eligibility (being outside an urbanized area as defined by the US Census) [70]. A list of host organizations and venues for events can be found at the study website [65].

To recruit sites, the study utilizes a community-based delivery model that we have described previously for successfully engaging underserved individuals in research [71]. Briefly, the model involves recruitment of experienced host organizations interested in facilitating community-based health events in partnership with the research team. The research team trains the hosts in using project tools and resources, and the host secures the venues and invites community members. At the event, community hosts introduce event attendees to the research team, who then invite the attendees to participate in the research trial, and then ensure that the study protocol is followed.

### Eligibility criteria {10}

#### Host inclusion and exclusion criterion

Potential hosts from the applicant pool are interviewed by the research team to explore their community connections, capabilities for hosting an event, and experience working with underserved populations. At least two research team members conduct each host interview, which are then reviewed and discussed at weekly team meetings prior to final selection of hosts. Hosts who provide informed consent complete a post-training feedback questionnaire, thereby allowing information from hosts to be included in the research results. Consented hosts also participate in a post-event qualitative research interview examining implementation. Inclusion criterion for the hosts is acceptance into the project and willingness to participate in the survey and phone interview. Hosts are excluded if they do not agree to the terms of the project. Hosts that are excluded from the project or cannot successfully complete an event will be interviewed (if willing) about their barriers.

#### Participant inclusion and exclusion criterion

Event attendees are eligible for inclusion in the research if they are:

1. Adults (≥18 years old)
2. Able to speak the language used at the event (English or Spanish), as determined by self-report
3. Able to read in English or Spanish, as determined by self-report

Participants are not eligible if they self-report difficulty hearing or communicating with others or have cognitive impairment (determined as inability to provide informed consent).

### Who will take informed consent? {26a}

Only research assistants (not the community event hosts) obtain in-person verbal informed consent, explain research procedures, and collect data. Event attendees who decline to provide informed consent are invited to stay and enjoy the community event, absent the research questionnaires and follow-up activities. Research decliners are asked to complete a brief de-identified survey to capture their demographic characteristics and reasons for declining. For the post-training host survey, hosts provide implied consent after reviewing a summary explanation of research and completing the survey. Additional verbal informed consent for host interviews is obtained by phone by research assistants prior to the qualitative host interview.

### Additional consent provisions for collection and use of participant data and biological specimens {26b}

Participant data will not be used for ancillary study, and no biological specimens are being collected.

## Interventions

### Explanation for the choice of comparators {6b}

To test efficacy of the *Hello* game, we selected *The Conversation Project Starter Guide* as a comparison because it is one of the most widely promoted and disseminated ACP tools nationwide. This starter guide is available for free online, and, like the *Hello* game, it is (a) approximately 60 min in duration; (b) does not require involvement of a healthcare provider or professional; and (c) uses open-ended prompts to promote discussing end-of-life issues with loved ones by having participants silently write down thoughts and share them with a group. *Hello* is different from *The Conversation Project Starter Guide* in that it includes elements of gamification, such as chips, game rules, and a “winner” at the end, whereas *The Conversation Project Starter Guide* structure includes questions with Likert scale-based response options. The *TableTopics* game is similar in structure to the *Hello* game but differs in that it is not related to ACP or end-of-life decision-making. It was selected as an attention control arm because of its functional similarity to the *Hello* game.

For all events and arms, participants are seated at tables in groups of 4-5. Our pilot studies showed that this number permits diversity of views that facilitate rich discussion and sharing. Hosts are provided with similar scripting across all arms to introduce the event activities. Attendees then either play *Hello* (Arm 1), discuss and complete *The Conversation Project Starter Guide* (Arm 2), or play *TableTopics* (Arm 3) for up to 60 min. Hosts and research assistants circulate to assist participants as needed.

Events in Hispanic communities will be facilitated in Spanish. All other community events will be facilitated in English. Regardless of the preferred or primary language spoken at the events, both English and Spanish materials are available at all events.

### Intervention description {11a}

#### Arm 1: The Hello Game

Commercially available, *Hello* [56] is a serious game that consists of 32 questions prompting players to share their values, goals, and beliefs about end-of-life issues. The creators developed the questions following interviews with palliative care clinicians, hospice nurses, and funeral directors, and then revised them through a series of focus groups with >100 patients/caregivers from diverse backgrounds. With permission, our team developed a rating scale and selected the most highly rated questions for use in our research. We then ordered the questions with attention to their emotional depth (to ensure variation in “heavy” and “light” questions) and breadth of content. The question order has been published to promote reproducibility [61, 62], and we use our modified question set for all research. At the event, players are provided the *Hello* question booklet and game chips. Hosts outline the rules: a player reads aloud the first question; players individually write down their answers; and then players take turns sharing their answers with the group (or opt to pass). Players’ answers typically prompt others to respond or ask questions, so what began as “reading an answer” evolves into a free-flowing conversation. An example question is “*In order to provide you with the best care possible, what 3 non-medical facts should your doctor know about you?*” Players control how long they share, what they share, and when they are ready to proceed to the next question. During the conversation, players may choose to acknowledge others who shared a particularly thoughtful, poignant, or even funny comment by giving them a chip. In accordance with game design theory [72] and also to promote lighthearted competition, a “winner” is named at the end of the game. A pre-game coin flip determines whether the player with the most chips “wins” the game (“heads”), or the player with the fewest chips “wins” the game (“tails”). However, to add intrigue to the game experience, the outcome of the coin flip is not revealed until the game ends.

#### Arm 2: The Conversation Project Starter Guide

The *Conversation Project* is a widely utilized public engagement initiative launched in 2010 and in collaboration with the Institute for Health Improvement and a panel of expert advisors and ACP advocates. The goal of *The Conversation Project* is to help individuals talk about their end-of-life wishes to increase the likelihood that these wishes are followed at end-of-life [73]. For the trial, we will use the *Conversation Project*’s 11-page workbook, which has open-ended prompts to consider one’s values and preferences for end-of-life care, whom to talk with about one’s wishes, and suggestions on how to do so. It also prompts participants to rank priorities on a 5-point scale (e.g., What are your concerns about treatment? 1= I’m worried I won’t get enough care, 5= I’m worried I’ll get overly aggressive care). The *Conversation Project* website provides resources for running a community event using *The Conversation Project Starter Kit*, a 23-page manual (“*Coaching the Conversation – A Guide to Facilitating Conversation Groups*”) that includes details on hosting a community-based program.

#### Arm 3: Placebo/Attention Control (TableTopics)

For use as an attention control, participants play a popular, commercially available game (*TableTopics*) [74] that consists of question cards to prompt conversations (e.g., “*What do you love about your hometown?*”). Participants have identical game instructions and procedures to *Hello* (i.e., using chips, writing down answers, taking turns/sharing). Questions that could possibly result in players discussing end-of-life issues are removed from the standard *TableTopics* deck to avoid contamination (e.g., “*If you only had five more years to live would you change anything about your life?*”). Like the interventional groups, at the end of the event, participants receive standard ACP materials. Community sites randomized to this group are approached after completion of the trial and are offered the opportunity to host an ACP community event using (per their choice) either *Hello* or *The Conversation Project Starter Guide*.

#### All arms: resource distribution and closing remarks

All participants in all groups receive standard, written ACP materials that include a paper AD and access to a paper ACP decision aid [75, 76]. Additionally, the *Start Simple with MyPlate Today* brochure (created by the US Department of Agriculture and US Department of Health and Human Services covering nutrition and healthy eating topics) is provided to participants in all arms. Hosts follow a script encouraging participants to review the nutrition and ACP materials, discuss their wishes with loved ones or clinicians, and complete ADs.

The trial team has no financial interest in any of the tools tested in the trial. All three companies/organizations provided permission to use their tool and had input into how the tool should be used in the trial to maintain fidelity to the product to the extent possible. The organizations also provided video and written materials for the project team to use during training of hosts and research assistants.

### Criteria for discontinuing or modifying allocated interventions {11b}

All hosts, research assistants, and other individuals assisting at the event are required to participate in a training webinar. This webinar explains the study protocol and the caveat that any participant may cease to participate upon request for any reason. An expert in grief counseling prepares research assistants to observe significant psychological distress due to the topic, and participants are offered the opportunity to pause or cease their participation in the intervention. The host training includes how to recognize the signs of psychological distress, including red flags, such as sudden and lasting changes in demeanor, crying that is interfering with communication, or leaving the room. A grief expert is available to assist hosts by phone as needed should an adverse event occur.

### Strategies to improve adherence to interventions {11c}

All hosts receive 4 h of standardized trainings plus one-on-one meetings with research assistants as they prepare their events. These trainings outline host responsibilities, which include facilitating the event, framing the topic, and introducing the research team to their communities during their event. The trainings cover how to carry out these responsibilities in a manner consistent with research standards. Although hosts will not conduct any research procedures, they still receive training on research ethics, privacy, confidentiality, and cultural sensitivity to ensure no breaches in research ethics occur. Host training topics include study overview and research ethics; hosting the event; arranging/marketing the event; and recognizing/responding to psychological distress.

To maintain the highest standards of fidelity and procedural rigor, a research assistant works one-on-one with hosts prior to the event to facilitate logistics and then travels to the site to oversee the event. The research assistants follow detailed procedure manuals developed from pilot work. Research assistants maintain detailed documentation regarding event circumstances, including when any participants leave an event early, complete surveys out of order, or other protocol deviations.

### Relevant concomitant care permitted or prohibited during the trial {11d}

To avoid contamination, hosts are instructed not to hold additional ACP events in their community in the 3 months preceding, and 6 months following the game event.

### Provisions for post-trial care {30}

Although in pilot studies there were no adverse events, in the event that a participant exhibits psychological distress during the event, a grief expert will be available to help connect them with community-based resources or a national grief resource if needed. After completing the study, sites randomized to the control arm are offered the opportunity to host an ACP community event using (per their choice) either *Hello* or *The Conversation Project Starter Guide* and made aware of whether one intervention was found to be more effective than the other.

### Outcomes {12}

Main outcome measures are discussed in detail below and are grouped according to the measured constructs: ACP, communication quality, impact of sociocultural environment on ACP, implementation and acceptability, and other (e.g., contamination, confounders). The full list of study measures and timelines are reported in Table 1.

**Table 1:** Study measures’ psychometrics, timeline, and analytic purpose

Instrument name and method	Items and duration	Psychometrics	Timeline				Purpose of variable and analysis plan
			Baseline	Immediately post-event	2–4 weeks post-event (phone call)	6 months post-event (phone call)	
Demographics^a^ (QUANT)	18q, 10 min	N/A	X				Baseline covariates
Healthcare System Distrust Scale [77] (QUANT)	9 q; 5 min	Values subscale= Cronbach alpha of 0.73; Competence subscale =Cronbach’s alpha of 0.77 (similar in African Americans and Whites)	X				
Experience and comfort with games (QUANT)	4 q; 2 min	N/A	X				
Previous exposure to ACP interventions and/or advance directives (QUANT)	6 q; 5 min	N/A		X^b^		X	Baseline covariates; Possible time-dependent covariates; contamination assessment
ACP Engagement Survey [78] (4-item version) (QUANT)	4 q; 3 min	Cronbach’s alpha 0.84; average 5-point scores were higher for people who engaged in prior planning (*P* < 0.001); detects change in response (0.4 points) to an ACP intervention. *p* < 0.001)	X			X	Secondary outcome
Acceptability of Intervention (QUANT)	3 q; 2 min	N/A		X			Descriptive
Conversation Satisfaction [79, 80] (QUANT)	8 q; 5 min	Reliability coefficient range .90–.97 reliabilities, validity coefficients up to .87 via nonverbal assessment scales		X			
Communication Quality Assessment [81, 82] Data transformation (QUAL to QUANT)	Audio recordings of ACP conversations	Intraclass coefficient range.73–.89, Cronbach alpha .69 to .89		During intervention			Compare conversation quality between interventions; mediation analyses
Experience and Perceptions of Intervention; Sociocultural environment; Adverse Events (QUAL participant Interview)	30 min	Interview guide follows NIH Best Practices for Rigor, Reliability			X		Assess adverse events; explores cultural norms related healthcare/ACP; empirical phenomenological analysis
Implementation and Sustainability Outcomes (QUAL host Interview*)*	30 min	Interview guide follows NIH Best Practices for Rigor, Reliability			X		Provide implementation data related to inner setting and intervention characteristics; conventional content analysis
AD Completion; “Other ACP Behavior” (QUANT)	6-26 q (depending on branching); 10 min	N/A				X	Primary and secondary outcomes
Impact of Sociocultural environment on behavior; Impact of Intervention on medical decision-making (QUAL participant Interview)	30 min	Interview guide follows NIH Best Practices for Rigor, Reliability				X	Explore how sociocultural environment impacts the ACP experience; empirical, phenomenological analysis; binary and ordinal logistic regression (via data transformation)

Two surveys were removed from protocol after three events due to survey burdens on participants (social support questionnaire and ACP values and beliefs)

^a^Includes race, ethnicity, gender, age, education, income, SES, religiosity, health status, marital status, experience with health decisions, medical decision maker presence at event, etc.

^b^Previous experiences with ACP questionnaire was moved to post-intervention timepoint after completing three events to reduce pre-intervention survey burden on participants

#### Outcome measures related to advance care planning

##### Primary outcome (AD completion)

The primary outcome for the trial is completion of a visually verified advance directive within 6 months post-event. We will visually verify ADs to reduce the likelihood of reporting bias inherent in self-report. To visually verify ADs, participants may choose one of the following strategies to securely share their signature page with the research assistant: mail return of a tear-out page from the AD via pre-paid envelope; contact the project notary who will notarize the AD and inform the project team; utilize simple “one click” encrypted texts or email links using REDCap for uploading images of AD signature pages; utilize host resources (i.e., scanners, fax machines); or show the completed document to the event host or research assistant using HIPAA-compliant video conferencing (Zoom). Project notaries will be available free of charge to participants who will confirm with the project team if they wish to notarize a form.

##### Secondary outcomes (Other ACP Behaviors and ACP Engagement)

During the 6-month follow-up, phone call participants are asked if they had engaged in additional ACP behaviors as measured by a questionnaire developed during our pilot studies [64]. Behaviors assessed include completing, updating/changing, re-reading/reviewing, storing, sharing, or notarizing AD documents; having conversations about end-of-life wishes or medical decision-making with loved ones, friends, family, and/or healthcare providers; researching information about nursing homes or long-term care, insurance, hospice or palliative care, or funeral planning; and document notarization. To measure readiness, as conceptualized by the trans-theoretical model and social cognitive theory, we are using the 4-item validated *ACP Engagement Survey* [78].

#### Secondary outcome measures related to communication

##### Communication Quality Analysis

During the two ACP interventions, two tables per event are selected by lottery for audio recordings of the intervention conversations. Participants preferring not to be audio-recorded are permitted to move tables. Audio recordings are transcribed verbatim for Communication Quality Analysis (CQA). CQA is a rigorous coding method for assessing clinical communication quality and is grounded in the Multiple Goals Theory of Communication [81–85]. Briefly, Multiple Goals Theory is a framework that defines high-quality communication as the extent to which three broad communication goals are achieved: (1) task goals (completing a task), (2) relational goals (maintaining healthy relationships with others), and (3) identity goals (managing self-presentation) [83]. When all three goals are attended to simultaneously, high-quality communication is said to have occurred. When any of the goals is ignored, lower-quality communication results [86]. CQA uniquely accounts for *how and for what purpose* something is said [82, 86, 87], whereas most of the commonly used measures of conversation quality base their ratings on *whether* something is said, using frequency counts of communication tasks or behaviors as a marker of high-quality communication [82, 86]. In contrast, CQA assesses the quality of conversation based on contextual elements (unaccounted for by other assessments) rather than the mere presence/absence of specific communication behaviors. Advantages of CQA over traditional methods include its ability to (1) measure quality using multiple conversational goals rather than mere frequency of behaviors; (2) account for the competing priorities that arise during real conversation; and (3) acknowledge contextual factors impact communication quality [86].

The CQA method involves coders reviewing audio recordings while following verbatim transcripts. For every 5-min interval, each coder assigns scores for each of 6 communication quality domains using procedures described below. Investigators trained in CQA train coders using published procedures [82] and several training modules. Coders (3 per conversation) then independently code several conversations and subsequently engage in group discussion until acceptable interrater reliability is achieved for all domains (intraclass *r* coefficient > 0.65) [88]. To ensure consistency between coders and to prevent coder drift, frequent reliability checks are performed. The 25-page CQA manual includes all coding rules, procedures, definitions, and examples of coding.

For each of the three goals (task, identity, and relational), coders provide scores for 2 domains per goal (Table 2). For each of the 6 domains, a score is assigned after every 5 min of conversation. Each domain is scored on a 7-point Likert scale, where 1 = lowest quality and 7=highest quality. As such, 6 scores are assigned every 5 min. If a participant is silent or has negligible contribution, a non-contributory code is assigned for that time segment.

**Table 2:** Conversation Quality Analysis Codebook: abbreviated definitions of domains

Goal	Domain	Brief definition of domains (full definitions below)
Task	*Content*	Discussing clinically relevant topics (e.g., attending to the clinical task); explaining medical options, testing, or treatments; exploring values/beliefs relevant to medical options; providing discrete directions for care; elaborating on reasons for recommendations
	*Engagement*	Paying attention, tracking with the conversation, asking others to elaborate on answers, exploring others’ points of view, trying to figure out what something means; elaborating on viewpoints; being direct and confident in discussing issues (not avoiding)
Relational	*Emotion*	Expressing vulnerability or intense emotions, providing emotional support, offering compassion and sensitivity when disclosing bad news, expressing empathy, disclosing personal experiences and thoughts, discussing hardships
	*Relationships*	Working hard to establish rapport, affirming the value of relationships, showing concern for others, expressing compassion or empathy, affirming value of involving family/friends in tough decisions
Identity	*Face*	Affirming others’ values or beliefs, listening with intent to understand, expressing a wish to honor others’ wishes, acknowledging others’ personalities in an affirming way, considering impact of decisions on others
	*Accommodation*	Tailoring to the other person’s communication needs or style; avoiding use of oversimplified speech patterns (like talking slowly, using simple words and grammar, using careful articulation); exaggerating intonation (like using a higher pitch or an overly familiar tone); showing appropriate sensitivity to the other person’s needs or questions; considering what the other person says; responding appropriately to the other person’s concerns; not interrupting; listening well; avoiding being scripted or robotic

**Fig. 2:**
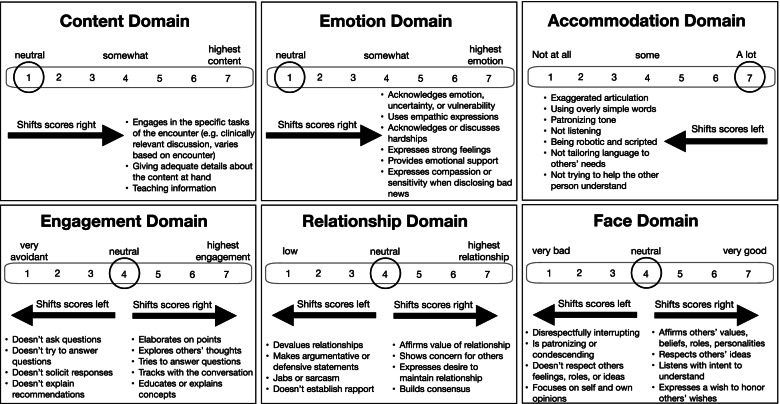
Overview of the Communication Quality Analysis coding procedures. A domain score is calculated for each of the six communication quality domains. As participants meet the definitions of each domain, the scores are increased. A domain score is assigned every 5 min for each of the six domains. All domains are scored 1–7 with “7” being the highest-quality score

Figure 2 demonstrates how coders translate the codebook into the numeric scores. To aid in calibration, coders begin at the neutral score for each domain and scores are increased (or decreased) as interactants engage in high- or low-quality conversation (as defined by criteria laid out in the codebook). As conversation ebbs and flows, coders increase or decrease scores so that at the end of each 5-min segment, the score represents the overall quality of the full 5-min increment. The neutral starting point varies based on domain (“1” for emotion, content, “4” for engagement, relationship, and face, and “7” for accommodation) for conceptual reasons—it is most sensible for coders to begin at a neutral midpoint for the engagement, relationship, and face domains and move up or down, given that the features of these domains can be present or absent. For other domains, such as “emotion” or “content,” it is more sensible for coders to move in a single direction as features of that domain are expressed. During measure development, these variations in starting (neutral) points maximized interrater reliability.

CQA empowers coders to serve as cultural informants rather than simply “detectors” of communication. This is done by allowing coders to increase or decrease scores following their instincts and drawing upon in situ cultural knowledge to integrate the affective intensity and frequency of behavior during communication. This means that the coders account for the “way” in which something is said, and adjust scores based on how the communication comes across (i.e., was received/interpreted) as opposed to sticking only to the verbatim discourse (i.e., whether something was said or not). This methodology provides an analysis that includes greater naturalistic accuracy than is afforded by coding systems that separate the frequency and intensity of discursive features [89].

**Fig. 3:**
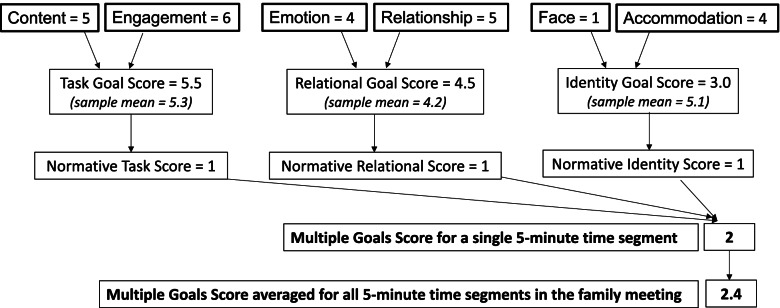
Calculation of the multiple goals score. The multiple goals score is a summative measure of all 6 quality domains that is reported in a single “breadth” score. To calculate the multiple goals score, each of the six domain scores are combined into three goals scores (task, relational, and identity goals). For each of these three goals, a normative score of “0” or “1” is assigned based on whether the score is above or below the sample mean. The MGS score is calculated for each time segment by taking the sum of each of the three normative goals scores (resulting a range from 0 to 3). Once the MGS score is calculated for each 5-min time interval, scores are then averaged across time intervals

Coders assign a score for each domain for each of the *Hello* questions, or every 5 min for *The Conversation Project Starter Guide* conversations. Domain scores are then used to calculate a single score, the Multiple Goals Score (Fig. 3), which incorporates all individual domain scores into a single ordinal score (0–3) that is then averaged across each time interval to reflect overall conversation quality.

#### Secondary outcomes related to the impact of sociocultural environment on ACP

##### Two to four weeks post-intervention semi-structured qualitative interview

Participants from interventional arms are purposively sampled (*n*=100 per arm; total *n*=200) based on interventional arm, race/ethnicity, gender, education, and whether they have a pre-event AD. Interviews explore sociocultural influences as they relate to the ACP experience during the event, potential adverse events from ACP, and perceptions about the host organization.

##### Six months post-intervention semi-structured qualitative interview

While all participants are contacted to complete the 6-month quantitative questionnaires, 200 participants are purposively sampled based on interventional arm, race/ethnicity, and gender for participation in a qualitative semi-structured interview (100 from each interventional group). Qualitative data are collected after quantitative data collection is complete. The interview explores the impact of the sociocultural environment on ACP behaviors or medical decision-making, motivations for participants to complete ACP (or barriers to doing so), and whether the sociocultural environment (i.e., race/ethnicity, community norms, social networks) played a role in behavior. Participants are asked whether any health situation involving medical decision-making occurred, and if so, how the event may have affected that experience.

#### Secondary outcome measures related to intervention implementation

##### Participant acceptability data

Intervention acceptability

This 3-item questionnaire assesses (immediately post-intervention) participants’ impressions about the intervention (including burden).

Conversation satisfaction

The communication satisfaction questionnaire is a validated, 8-item questionnaire that is correlated with likelihood that conversational goals are met [79]. It will be administered immediately post-intervention.

##### Host implementation data

Post-webinar training host questionnaire

This 12-item mixed-method questionnaire was created for the project to assess host perceptions about how well the training sessions and materials prepared hosts for running an event.

Host semi-structured interviews on implementation and sustainability

Hosts from both interventional groups (*n*=60) are interviewed to explore aspects of the intervention characteristics and inner setting; 2 domains from the Consolidated Framework for Implementation Research (CFIR) that are relevant to the project [90]. Intervention characteristics that are explored include complexity, adaptability, relative advantage, and costs. For the inner setting domain, interviews focus on compatibility as well as networks and communications. This guide will be built using the CFIR Interview Guide Tool, an online tool maintained by the developers of the CFIR that offers researchers a menu of customizable semi-structured questions for each CFIR domain and construct.

#### Measures to assess contamination and other potential confounders

A 6-item questionnaire will be administered at the 6-month follow-up phone call to assess for potential exposures or confounders to other ACP interventions or to identify potential intervening events that could impact ACP behavioral outcomes (e.g., unintended ACP intervention or other conversation activity exposures, hospitalizations, changes in personal health status, experienced births/deaths). These variables are accounted for during analyses.

### Participant timeline {13}

The study timeline and measures are shown in Table 1.

### Sample size {14}

Sample size calculation involved a computer simulation study (SAS PROC Power) with a nonlinear mixed-effects model to determine the required number of recruitment sites and participants per site for detecting a difference between 50% AD completion rate for those in the *Hello* group and 35% AD completion rate for those in *The Conversation Project Starter Guide* group, with 80% power using a two-sided, 0.05 significance level test. In our pilot work, we observed a 43% AD completion rate and anticipate higher rates in this study due to additional steps taken to reduce attrition [64]. Under these design assumptions, the study has 99% statistical power to compare the “*Hello*” to the Placebo/Attention Control group, where we expect AD completion rates of 50 and 25%, respectively. There is 82% statistical power to compare *The Conversation Project Starter Guide* arm to the Placebo/Attention Control arm, where we expect AD completion rates of 35 and 25%, respectively. These analyses account for a conservative intra-cluster correlation coefficient of 0.20 and a conservative 30% attrition within each of the 75 sites based on pilot data from a similar population {Hayes, 2009 #1259}. Should a site drop out (<10% in the pilot) [64], a new site will be recruited with similar characteristics.

### Recruitment {15}

Our approach for engaging underserved populations in ACP uses a community-based delivery model that leverages existing sociocultural networks within Black and Hispanic communities to partner with existing and deeply respected social institutions (e.g., places of worship, community centers) [64, 71]. Host organizations are recruited in partnership with the Hospice Foundation of America (HFA) using strategies that utilize their list-servs, networks, informational webinars, social media, and press releases announcing the opportunity for community organization participation in the trial. HFA’s vast network includes a wide range of community-based organizations: end-of-life care coalitions, colleges and universities, hospitals, hospices, libraries, funeral homes, and places of worship. Interested hosts are directed to the project website [65] to complete an application that describes the characteristics of their underserved communities, their plans for venue bookings and marketing, details of their community connections, and previous experiences with planning and hosting community events in underserved communities. Hosts secure their event venues (including a signed venue letter of support), promote events through social networks, and invite potential participants to attend (using IRB-approved materials—flyers, newsletters, radio ads, emails, social media postings, and announcements). Hosts are permitted to personalize some aspects of the materials by including their organization information and logos. Since communities under study vary significantly regarding population, demographics, culture, etc., we work with individual sites to adapt the materials as needed (with IRB approval).

Hosts are required to collect RSVP information to include the number of attendees within 48 h of the event to help ensure that turnout will be strong enough to warrant the research team travel to the site. Research assistants travel to each host site and facilitate attendees’ participation in the research aspects on the day of the event. Attendees are eligible if they participate in the community event and meet additional criteria via using self-report. After welcoming attendees to the event, the research assistant describes the research, inclusion and exclusion criteria to potential participants. Participants complete a simple form confirming that they meet eligibility criterion. Then the research assistants review a Summary Explanation of Research with the group. Time is allotted for questions, and attendees provide verbal informed consent before participating.

## Assignment of interventions: allocation

### Sequence generation {16a}

Randomization strata considered combinations of geographical rurality, Spanish or English language spoken, and race (Table 3). These strata were selected because race/ethnicity is known to affect engagement in ACP, and rural status is known to limit access to healthcare resources, reducing the likelihood that ACP occurs.

**Table 3:** Targeted recruitment × randomization strata for *n*=75 sites

Strata	***Intervention Hello***	***Intervention CP***	***Control TableTopics***	Total
Urban Non-Hispanic Black	6	6	3	**15**
Urban Non-Hispanic White	6	6	3	**15**
Hispanic (Spanish-speaking)	6	6	3	**15**
Rural Non-Hispanic Black/African American	6	6	3	**15**
Rural Non-Hispanic White	6	6	3	**15**
**Total**	**30**	**30**	**15**	**75**

The randomization sequence was created using a randomization with permuted blocks of fixed size to accommodate a 2:2:1 allocation ratio via SAS software Version 9.4 PROC PLAN. The randomization sequence was generated to accommodate the randomization of 75 sites with 5 strata, with 15 sites per strata.

### Concealment mechanism {16b*}*

The primary concealment mechanism utilized for this trial relies predominantly on the randomization module (application) of the REDCap database system (HIPAA-compliant database platform) which allows maintenance and control over user rights and role access to the trial database [91]. Only the statisticians and data manager have access to the complete sequence list that REDCap uses to complete the host randomizations. Once a host is eligible to be randomized and the randomization button is selected, an unblinded researcher (lead Project Manager or member of the coordinating team) is informed of arm assignment. The team then coordinates the host’s remaining arm-specific training and is responsible for packing and transporting all arm-specific materials to the event site. Additional masking is achieved using a randomized letters (A, B, or C) to mask intervention assignment. Only the lead Project Manager has access to the linkage.

### Implementation {16c}

The lead Project Manager randomizes the host using the REDCap database and communicates the arm assignment to the research assistants involved in implementing and carrying out the events. All other study team members not involved in event facilitation tasks related to the host’s assigned allocation (including the PI, co-investigators, and statisticians) remain blinded to the greatest extent allowable. In the unforeseen circumstance that a randomized host is ultimately unable to fulfil their commitment to the project in hosting an event for their strata, the host record is deleted from the database to eliminate their previously filled allocation slot within the randomization sequence. This allows an alternate host of similar strata to eventually “replace” the allocation previously assigned to the dropout host. If needed, a host can be removed and re-randomized if they are agreeable to switching strata and receiving a new randomized assignment (see “Discussion”).

## Assignment of interventions: blinding

### Who will be blinded {17a}

After arm allocations are assigned and disclosed through REDCap, the coordinating team, event hosts, and study participants/event attendees all eventually become aware of the host/event allocation assignment. The PI, co-investigators, and statisticians remain blinded throughout the trial to the extent possible. Thus, only the research assistants (coordinating team) work with hosts during event planning. Questions and issues are brought to the PI and remainder of the research team at weekly team meetings in ways that maintain blinding. Hosts are made aware of their arm assignment so they can advertise, market, and invite participants appropriately.

Due to the study design and behavioral intervention, a single-blind is not always possible. The trial employs a 2:2:1 allocation ratio and facilitates certain study procedures that are only applicable to the two intervention arms (inapplicable to the attention control). Because of the nature of this study, it is possible for unblinding instances to occur for the control arm; however, the aforementioned procedures are in place to prevent this from occurring

. Additionally, communication with hosts or media coverage of community events may inadvertently unblind team members. Research assistants screen materials and emails to the extent possible to avoid these occurrences.

For additional rigor and best practice, research team members who collect or analyze outcome data at the 6-month follow-up time point are blinded wherever practical. Although it is possible that the participants may unintentionally unblind the interviewer during the qualitative portion of the interview, quantitative data (including the primary outcome) are collected first to prevent potential bias in quantitative data collection.

### Procedure for unblinding if needed {17b}

If unblinding becomes necessary due to logistical issues related to travel, event preparations, or data collection procedures, the PI will delegate an appropriate team member to become unblinded as necessary to assist the research staff. Standard decision-making and problem-solving over the course of the trial is unlikely to require unblinding unless absolutely necessary for resolution.

## Data collection and management

### Plans for assessment and collection of outcomes {18a}

All data are collected during events using paper forms (or audio recordings of ACP conversations in the two interventional arms), and participants have their choice of completing forms in English or Spanish. Reading assistants circulate to assist participants if necessary and monitor form completion to minimize unintentional missing data or skipped items/pages. Reliability and validity data for study instruments are provided in Table 1. Data collection forms can be requested from the corresponding author. Research assistants collect data by phone or video conference based on participant preferences and audio-recorded to allow quality checks and transcription of qualitative interviews. Data collected via phone or video conference at the 6-month follow-up time point are entered directly into REDCap during the interview to ensure the interviewers have access to real-time database triggers, data quality checks, branching logic, reminders, etc. Visual verification of advance directives is achieved using one of the following strategies described above (see “Outcomes {12}”).

### Plans to promote participant retention and complete follow-up {18b}

Several strategies are used to maximize participant retention during the follow-up period, including collecting detailed contact preferences, sending reminder postcards and text messages in advance of the follow-up calls, distributing refrigerator magnets with the project contact information, and use of a recognizable project phone number for caller ID. Participant stipends are provided after completion of follow-up interviews. Up to five attempts at contact are made prior to participants being considered lost to follow-up.

### Data management [67]

The Penn State Public Health Sciences Data Management Unit monitors the data. Research assistants review instruments in real time to the extent possible to identify missed items that then will be pursued to render the resultant database as complete as possible. Branching logic, range checks, and quality rules are programmed to minimize potential errors in data entry. During follow-up interviews, assistants enter the data directly into the REDCap database and interviews are recorded for periodic, random data checks by the project manager.

### Confidentiality {27}

Confidentiality of written data is maintained by de-identified participant code numbers. Only the unblinded research assistants have access to these linkage codes. Trained research assistants maintain and secure all data during travel. Participants are reminded that verbally shared information at the event are to remain confidential, and to use their study numbers when identifying themselves during the recordings to the extent possible. Recordings are uploaded immediately from audio recorders to a secure, encrypted network, and devices are wiped of all audio data and all transcripts are de-identified. Secure file transfer systems and REDCap databases are used to minimize the risk of confidential or private data being obtained by non-study investigators. Paper data are maintained in locked offices and mailed data is marked as confidential and sent securely.

### Plans for collection, laboratory evaluation, and storage of biological specimens for genetic or molecular analysis in this trial/future use {33}

No biological specimens are being collected for this trial.

## Statistical methods

### Statistical methods for primary and secondary outcomes {20a}

#### Quantitative analyses

The primary outcome of AD completion, as measured within the 6 months after the trial event, will be analyzed as a binary response. This primary outcome is determined by visual verification and is defined by dated signatures post-event (be it completion of a new written AD, or the official updating or changing of an existing AD). We will invoke a nonlinear mixed-effects model with a logit link function [92]. The model will consider (1) fixed effects for the randomized group, participant race, ethnicity, gender, religiosity, health status, decision-making experience, healthcare distrust, game playing experience, and previous exposure to ACP interventions, and (2) random effects for event site and presence of social support at the event (specifically, attending the event with their medical decision maker). Based on the analytical results, we will construct odds ratios, along with corresponding 95% confidence intervals, comparing the interventions to each other, and each of them to the control.

Other ACP behaviors and the change in readiness (ACP Engagement Survey scores) will be analyzed as secondary outcomes similarly using the nonlinear mixed-effects model described above, but with an ordinal logit link function. Secondary analyses will allow for the completion of other ACP behaviors to be “counted” and defined more broadly via self-report (without visual verification for actions or behaviors). We will also calculate proportions of those who submitted AD documentation (visual verification) out of all those who self-reported completion of “any” ACP behavior, as well as the confidence intervals for that proportion. To explore whether mediating variables (e.g., other potential motivators since the event, such as recent deaths, changes in health status, other ACP exposures; Table 1) affect completion of ACP behaviors, we will conduct a mediation analysis using a four-way decomposition that determines the controlled direct effects of the randomized arm, the reference arm × mediator interactions, the mediated interactions, and the pure indirect effects of the mediators [93].

We also plan to investigate the effect of the conversation structure (i.e., group) on the CQA variables via nonlinear mixed-effects models with ordinal logit link functions in similar fashion as described for the secondary analyses in Aim 1. We will apply mediation analyses described above [93] to investigate whether the CQA variables affect the outcomes of AD completion, other ACP Behaviors and change in score on the ACP Engagement Survey. SAS Version 9.4 (PROC NLMIXED and PROC CAUSALMED) will be used for analyses.

#### Qualitative analysis and mixed methods integration

The goal of the qualitative analysis is to learn whether and how the sociocultural environment contributes to participant perceptions and experiences during the interventions. These qualitative data will be transformed into categorical data for statistical comparisons across race/ethnicities and arms (a process called data transformation, see below) [66]. To ensure adequate power for these statistical comparisons, we will purposively sample and analyze 200 participants (100 per interventional arm). Collecting many similar cases increases rigor and bolsters trustworthiness of qualitative data [94]. To the extent possible, we will also sample based on participants’ gender, age, and scores on both the attitudes and the healthcare system distrust questionnaires in order to obtain diverse representation of the full study population so as to draw the most generalizable conclusions possible. Analysts will be blind to the sampling characteristics during coding.

To perform the qualitative analysis, we plan to use an empirical, phenomenological approach [95], which is useful when trying to understand individuals’ common, lived experiences regarding a phenomenon (i.e., ACP experiences since the event) [95]. Empirical phenomenology focuses on participants’ *description* of the experience [95]. Analysts will use standard qualitative procedures to analyze the data (i.e., bracketing, horizontalization, and textural description) [66, 96]. The result will be a robust description of the essence of participants’ lived experience performing ACP since the event. Qualitative software (MAXQDA) will be used to organize and code the data. Published criteria for methodological rigor of qualitative research will be used to attend to the truth value, applicability, consistency, and neutrality of our findings [97]. To evaluate the credibility (i.e., truth value) of the findings, a sub-set of participants from each group will be asked to review a summary of data to ensure accuracy (member checking) [66]. To bolster applicability of the findings to other contexts, rich/thick descriptions from the interviews will be obtained so that readers undergoing a similar experience may assess and compare experiences [98]. Consistency and dependability of data will be maintained by frequent interrater reliability analyses using intraclass correlation coefficients [94, 99]. Researchers will bracket biases and create an audit trail of coding decisions to maximize data neutrality [98].

For the 2–4-week host and participant interviews, we will use directed content analysis [95, 100]. Categories and codes will be assembled into a codebook and constant comparison method used [101]. Inter-coder reliability will be defined by an intraclass correlation coefficient of >0.7; discrepancies will be managed by discussion [99]. Final codes will be examined for themes.

### Interim analyses {21b}

No interim analyses are planned during the trial.

### Methods for additional analyses (e.g., subgroup analyses) {20b}

We will explore subgroup analyses via the nonlinear mixed-effects models described above for each of the five randomization strata. These subgroups will also be explored qualitatively. Depending upon the distribution of participants who attend an event (1) without any prior written AD, (2) with a written AD completed within 5 years of the event, or (3) with a written AD completed more than 5 years prior to the event, either subgroup or adjusted analyses could be explored. Adjusted analyses would allow for prior written AD status to be controlled for in primary and secondary analyses, and potentially for the assessment of a prior written AD status effect on the outcomes. Subgroup analyses would allow for an evaluation of the randomized arm effect in subsets of participants with varying levels of experiences with written ADs. Those who enroll in the trial with prior written ADs could potentially update or change their existing documents, while those without prior written ADs would be assessed for completion of a new written AD.

Themes derived from qualitative analyses will be used to contextualize trial results by data transformation, which involves coding emerging themes into discrete categories (quantitative variables) [66, 102–104]. By nature of the study design, these categories will be created based on findings from the qualitative analysis. An example might include a binary indicator of whether participants described negative experiences with the healthcare system as a barrier to completing an AD. In this example, we would use binary and ordinal logistic regression to determine whether participants who completed an AD were more or less likely to have a negative experience with the healthcare system. Statistical comparisons of transformed themes will be conducted based on race/ethnicity (Table 4) and arm (Table 5).

**Table 4:** Race/ethnicity analysis: planned purposive sampling and hypothetical joint display reporting mixed methods integration

ACP behavior	Race	Hypothetical common themes	Hypothetical arm-specific themes	Hypothetical conclusions from integration
Yes	Black (*n*=40)	• Values ACP conversations • Family important	• Experience with end-of-life decisions • *Family as support* system	Black participants who described *strong family support* were 5 times more likely than white participants to complete an ACP behavior (*p*<0.01) Regardless of racial/ethnic background, those who described *negative experiences* within healthcare were 5 times less likely to complete an ACP behavior (*p*<0.01)
	White (*n*=40)		• Positive experiences with healthcare	
	Hispanic (*n*=20)		• Community roles in decision-making	
No	Black (*n*=40)	• Distrust of healthcare system • Funeral planning important • Skeptical about the value of ACP	• Prior *negative experiences* within healthcare system	
	White (*n*=40)		• Strong social networks besides family • Prior *negative experiences* within healthcare system	
	Hispanic (*n*=20)		• Distrust of legal documents

**Table 5:** Arm analysis: planned purposive sampling and hypothetical joint display reporting mixed methods integration

ACP behavior	Arm	Hypothetical common themes	Hypothetical arm-specific themes	Hypothetical conclusions from mixed methods integration
Yes	*Hello* (*n*=40)	• Family is important • Community is important • Experience with end-of-life decisions	• Find *value in ACP* conversations • Religion important support system	Those who played the game were 5 times more likely to *find value and benefit of ACP* than those who used *The Conversation Project Starter Guide* (*p*<0.01) Those who participated in an ACP intervention were 5 times more likely to *find value and benefit of ACP* than those who were in the control arm (*p*<0.01)
	*The Conversation Project Starter Guide* (*n*=40)		• Prior *positive experiences* within healthcare system • Skeptical of the value of ACP	
	*TableTopics* (Control) (*n*=20)		• Prior *positive experiences* within healthcare system	
No	*Hello* (*n*=40)		• Find *value and benefit to ACP* conversations	
	*The Conversation Project Starter Guide* (*n*=40)		• Religion is an important support system	

### Methods in analysis to handle protocol non-adherence and any statistical methods to handle missing data {20c}

All statistical methods and approaches will follow intention-to-treat (ITT) principles, in that participants will be analyzed “as-randomized,” and all participants who attended event day activities (unless they formally dropped out or withdrew from the study) will be included in analyses. Contamination (reported additional ACP exposures, prior experience with activities in other arms, attending multiple events, etc.) will be captured and assessed to the greatest extent possible, and controlled for in analyses as necessary.

Further, missing data for the AD completion and ACP behavior outcomes at the primary endpoint will be handled using a conservative sensitivity analysis approach, which will assume that missing outcome data represents non-completed ADs or non-completed ACP behaviors, where applicable. Similarly for the change in readiness outcome, missing scores on the ACP Engagement Survey at the primary endpoint will be analyzed under the assumption that the participant did not experience any change in readiness from their assessment at baseline on the event day. For analyses that consider many covariates, patterns of completeness and the amount of missing data for each of those covariates will be evaluated to determine the most appropriate approach for handling missing data, such as multiple imputation.

### Plans to give access to the full protocol, participant-level data, and statistical code {31c}

De-identified data, protocol access, and statistical code are available upon reasonable request to the PI.

## Oversight and monitoring

### Composition of the coordinating center and trial steering committee {5d}

The trial project team, led by the principal investigator, consists of an interdisciplinary team of experts in advance care planning, end-of-life care, critical care, ethics, medical humanities, community engagement, health economics, statistics, hospice, implementation science, and public health. This full interdisciplinary trial team is supported by a team of two project managers, several research assistants, graduate students, and a marketing specialist. The project is also supported by a national grief expert.

### Composition of the data monitoring committee, its role and reporting structure {21a}

This minimal risk trial is overseen by a Safety Monitoring Committee (SMC) that includes a research ethicist and biostatistician with expertise in multi-center clinical research. A grief counselor also provides consultation to the SMC. The SMC convenes at least annually to provide a full review of recruitment data, data collection, and adverse events. The SMC works with the project team to review monitoring and regulatory reports annually. The PI is responsible for reporting all adverse events to the SMC, sponsor, and other necessary parties as described in the Data Safety Monitoring Plan (available upon request from the lead author). The SMC is independent from the sponsor and competing interests.

### Adverse event reporting and harms {22}

For this minimal risk trial, the most likely adverse event is psychological distress of participants, though we defined an adverse event as any untoward medical occurrence in a subject causally associated with participation in the clinical study. Any harms reported in association with participation in this trial will not be classified according to standardized criteria, as we anticipate adverse events to be very infrequent and minimal based on experiences from prior studies. In trial publications at the end of the study, we plan to report all adverse events determined to be possibly related to participating in the trial. Adverse events will be categorized as mild, moderate, or severe, along with their expectedness and seriousness. All hosts and research assistants are required to participate in a training webinar in which an international expert on grief discusses psychological distress. The webinar trains all research assistants and community hosts on recognizing the signs of psychological distress, including issues related to diversity, equity, and inclusion. The training highlights potential red flags for adverse events (for example, sudden and lasting changes in demeanor, crying that is interfering with communication, leaving the room). The grief consultant assists sites with strategies for referral to a local healthcare counselor and is available to assist hosts and/or participants as needed (by phone or video conference) should an adverse event occur. The PI is informed of any serious adverse event or unanticipated problem (regardless of grade, expectedness, or relatedness) as soon as they occur. Any adverse events that are spontaneously reported to the research assistants throughout the trial are then reported to the PI within 24 h of occurrence. The PI then notifies the SMC chair verbally within 24 h and generate a written report for the SMC within 72 h. The SMC annually reviews reports that include the number and types of adverse events per arm. The SMC statistician also reviews recruitment data and accrual trends to ensure recruitment pace is on track and will be responsible for overseeing attention to data integrity by reviewing reports from the team.

### Frequency and plans for auditing trial conduct {23}

The Penn State University Institutional Review Board (IRB) oversees the protection of human subjects in research and conducts continuing review of the trial. The SMC reviews data reports at least annually independently from the investigators and sponsor.

### Plans for communicating important protocol amendments to relevant parties (e.g., trial participants, ethical committees) {25}

All communication between the hosts and the single IRB occurs through the on-site research assistants, Project Manager, co-investigators, or PI. Hosts are required to submit any modifications or changes to marketing materials to the research assistant who reviews the materials with the study team and then submits them to the IRB for approval. All written venue agreements and reliance agreements are provided to the research team and then forwarded to the IRB. The PI and/or the Project Manager ensure that all agreements are in place, documents are maintained and renewed, and are in compliance with NIH and IRB policies.

### Dissemination plans {31a}

Findings will be presented to the scientific community via abstracts submitted to international or national scientific meetings and peer-reviewed publications. The team will disseminate results to community members, participants, and the public through press releases, a 10-page Community Brief to participating host sites for distribution within their communities, the project website [65], and other professional networks. Data will be made available in a public repository upon completion of the trial.

## Discussion

The Project Talk Trial is designed to provide data that comprises three studies in one by comparing *The Conversation Project Starter Guide* and the *Hello* game each to a control arm, as well as to each other. The trial will provide key outcomes to learn if either intervention helps motivate participants to complete an advance directive and will provide comparative efficacy data for the interventions. Further, the trial is designed to provide insight into potential mechanisms of action for the conversation activities by evaluating if sociocultural influences and the quality of communication triggered by the interventions mediates advance directive completion. Another strength of the Project Talk Trial is that it is the first community-based interventional trial (to our knowledge) to use visual verification of advance directives as an outcome rather than relying solely on self-report. These strengths bolster the rigor of measuring our primary outcome when compared with similar trials, and, because we will collect both self-reported and visually verified advance directive completion data, the trial data will allow us to assess the validity of using self-reported AD completion for future studies.

As with all clinical trials, there are and will be challenges to overcome. The COVID-19 pandemic causes substantial challenges with regard to scheduling community-based events amidst COVID-19-related public health concerns, travel concerns, and evolving CDC recommendations. The pandemic began prior to any Project Talk hosts being enrolled in the study, and therefore, our study team was able to adopt several strategies that will address the uncertainty of conducting a community-based trial amidst a public health crisis. These strategies include (1) allowing host organizations to join the project and host events on a rolling timeline that can accommodate differences in community spread across the USA; (2) incorporating flexibility for event delays and postponements of events; and (3) following both federal and local health guidelines for all events including provision of masks. Finally, while the pandemic impacted our project initiation timeline, we will leverage resources to double our efforts during spring and summer months when COVID-19 transmission is at its lowest to avoid further delays.

We also anticipate challenges related to community engagement research that will require hosts to move their event from one community venue to another, possibly including changes in their randomization strata. To minimize the effects of this challenge, we will randomize hosts after completing venue letters of support. Additionally, it is possible that attendees invited to the events will not “match” the strata classification of the event site since anyone can attend and participate in the events regardless of their race, ethnicity, zip code, socioeconomic status, etc.. We do not screen or turn away potential attendees at the door. To assure that we capture data from our target population, we will review demographic characteristics from each site prior to analysis to confirm that the anticipated population characteristics were met.

Upon completion of our first 3 events (1 Spanish-speaking and 2 English-speaking), we noticed that the pre-intervention study measures were taking twice as long as allotted in the protocol. Therefore, to minimize survey burden, two measures were removed from the protocol (an ACP values and beliefs questionnaire and a social support questionnaire). Additionally, one measure that collects prior experiences with medical decision-making was moved from pre- to post- activity to provide a better balance of questionnaires before and after the conversation activity.

Upon completion of the trial, we will have provided data to add to the evidence base behind various conversational ACP interventions, data to examine potential mechanisms of action for such ACP interventions, and qualitative data to better understand the sociocultural environment of how community-based ACP interventions are experienced by underserved populations. We will also provide important data for future researchers to learn if and whether visual verification of advance directives is necessary, or whether reliance upon self-reported outcomes is of comparable value.

### Trial status

Protocol version and date. Protocol version 7 dated 6/9/2022.

Host recruitment began April 12, 2021.

The first event with participant recruitment began March 19, 2022.

The trial is expected to run through June 30, 2025.

## Data Availability

All data and study materials are available upon reasonable request to the Principal Investigators.

## Abbreviations

ACP: Advance care planning
AD: Advance directive
CQA: Communication Quality Analysis
PI: Principal Investigator
SMC: Safety Monitoring Committee
DSMP: Data Safety Monitoring Plan
IRB: Institutional Review Board
NIH: National Institutes of Health
